# The enigmatic case of *Lipoptena* sp. in the Bosco della Mesola Nature Reserve (Italy)

**DOI:** 10.1111/mve.70002

**Published:** 2025-08-20

**Authors:** Federica Usai, Filippo Maria Dini, Ilaria Guarniero, Enrica Bellinello, Laura Stancampiano

**Affiliations:** ^1^ Department of Veterinary Medical Sciences University of Bologna Bologna Italy; ^2^ Research, Ecology and Environment Dimension (D.R.E.Am. Italia) Pistoia Italy

**Keywords:** NUMTs, *Cervus elaphus italicus*, COI, *Lipoptena andaluciensis*

## Abstract

Species belonging to the genus *Lipoptena* have received limited attention and have historically been subject to misidentifications. Recent records of *L. fortisetosa* in Europe have rekindled interest in these hippoboscids, leading to the discovery of a new species in Spain in 2024, which has been named *L. andaluciensis*. During an opportunistic sampling conducted in March 2023 and October 2024 on the Italian red deer (*Cervus elaphus italicus*), an Italian endemic subspecies, within the ancient relict lowland forest of the Bosco della Mesola Nature Reserve, hippoboscids morphologically identical to *L. andaluciensis* were collected and examined. This represents the first record of this species in Italy. In total, 257 specimens of *Lipoptena* were collected and analysed (161 in 2023 and 94 in 2024), all identified as *L. andaluciensis*. Additionally, *Lipoptena* specimens collected from fallow deer in 2007 within the same nature reserve, which had remained unidentified until now, were re‐examined. A total of 66 specimens were reassessed, of which 63 were morphologically identified as *L. andaluciensis* and 3 as *L. cervi*. Morphological identification of the 2023 and 2024 specimens was further confirmed through molecular analysis using COI as a barcode marker. Molecular analysis also revealed the presence of a nuclear copy of the COI gene (NUMTs) in the nuclear genome of *L. andaluciensis*. The discovery of *L. andaluciensis* in Spain and in Italy since 2007 raises the possibility that this species has a much broader distribution, particularly at lower altitudes and within a Mediterranean climatic zone. It is plausible that its presence has so far gone unnoticed or has been misidentified.

## Graphical abstract

- This is the first report of *Lipoptena andaluciensis* in Italy, confirmed morphologically and genetically.
- The presence of *L. andaluciensis* in Italy dates back many years; it was found on cervids in lowland habitats.
- A re‐examination of the previous report of the Italian *L. fortisetosa* is needed.

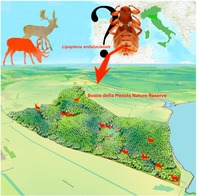

## INTRODUCTION

Deer keds (genus *Lipoptena*, family Hippoboscidae) are obligate blood‐feeding ectoparasites that shed their wings after finding a suitable host, preferably a cervid. Winged adults have limited flight capability and must quickly find a nearby host; thus, the movement of these insects is closely tied to the movement of their hosts. The loss of wings also ensures that these parasites remain closely associated with the selected individual host for their entire life, which can last several months (Haarløv, 1964).

*Lipoptena* genus has for a long time been overlooked by the scientific community probably because the host range for most species is restricted to wildlife (Bezerra‐Santos & Otranto, 2020). Moreover, the collection of these flies can be difficult due to their close association with their hosts. In Europe, limited research has been conducted on this genus until the recent report of the exotic species *Lipoptena fortisetosa*. This ked has drawn considerable attention due to its impact on various autochthonous mammals and its expanding distribution across Europe (González et al., 2024).

In Italy, until recently, the only species present was *Lipoptena cervi*, but recent studies by Andreani et al. (2019, 2021) have revealed the presence of *Lipoptena fortisetosa* in the regions of Tuscany and Emilia‐Romagna. The origin of *L. fortisetosa* lies in Japan, where its primary host is the sika deer (*Cervus nippon*). It was assumed that through the relocation of this ungulate, *L. fortisetosa* has gradually expanded its range, reaching Tuscany as its southernmost limit (Andreani et al., 2021; Kurina et al., 2019).

Rehbein (2021) highlights that *L. fortisetosa* has actually been present in Europe since the 1930s as revealed by the re‐examination of historical museum collections. These findings suggest that this species may have been mistakenly identified in the past as the closely related *L. cervi*, as also discussed by Mihalca et al. (2019). Similarly, given the difficulty of identifying these insects, it cannot be ruled out that the growing interest in *L. fortisetosa* has led to hasty reports and that some of the recent records of *L. fortisetosa* may also be the result of identification errors.

A thorough investigation of the *Lipoptena* genus would therefore be necessary, especially in light of the recent discovery of a new *Lipoptena* species for Europe by González et al. (2024). This was named *Lipoptena andaluciensis*, whose resemblance to *L. fortisetosa* is remarkable.

It would also be of particular interest to further investigate the distribution and habitat preferences of *Lipoptena* species, about which so little is known.

In a recent study based on the data collected by Andreani et al. (2021), Stancampiano et al. (2025) demonstrated that *L. fortisetosa* is preferentially distributed at low altitudes, even near anthropised areas, compared with *L. cervi*, which is more frequently found at high altitudes (above 600 m). This different geographical distribution, which would tend to minimise the competition between the two species, certainly deserves further investigation, especially in lowland forests inhabited by cervids.

The aim of this study is to investigate the presence of hippoboscids belonging to the genus *Lipoptena* in an environment of extreme naturalistic and ecological interest, such as the Bosco della Mesola Nature Reserve, located in the province of Ferrara (Emilia‐Romagna region, Italy). This forest represents one of the last and best‐preserved remnants of lowland forest in Italy. Its unique environment is a biodiversity hotspot in an area with an agricultural vocation, and it is home to the last population of the endemic Italian red deer (*Cervus elaphus italicus*), a population threatened by genetic isolation and the limited availability of food in a restricted habitat (Lovari & Nobili, 2010).

## MATERIALS AND METHODS

### Study area

**FIGURE 1:**
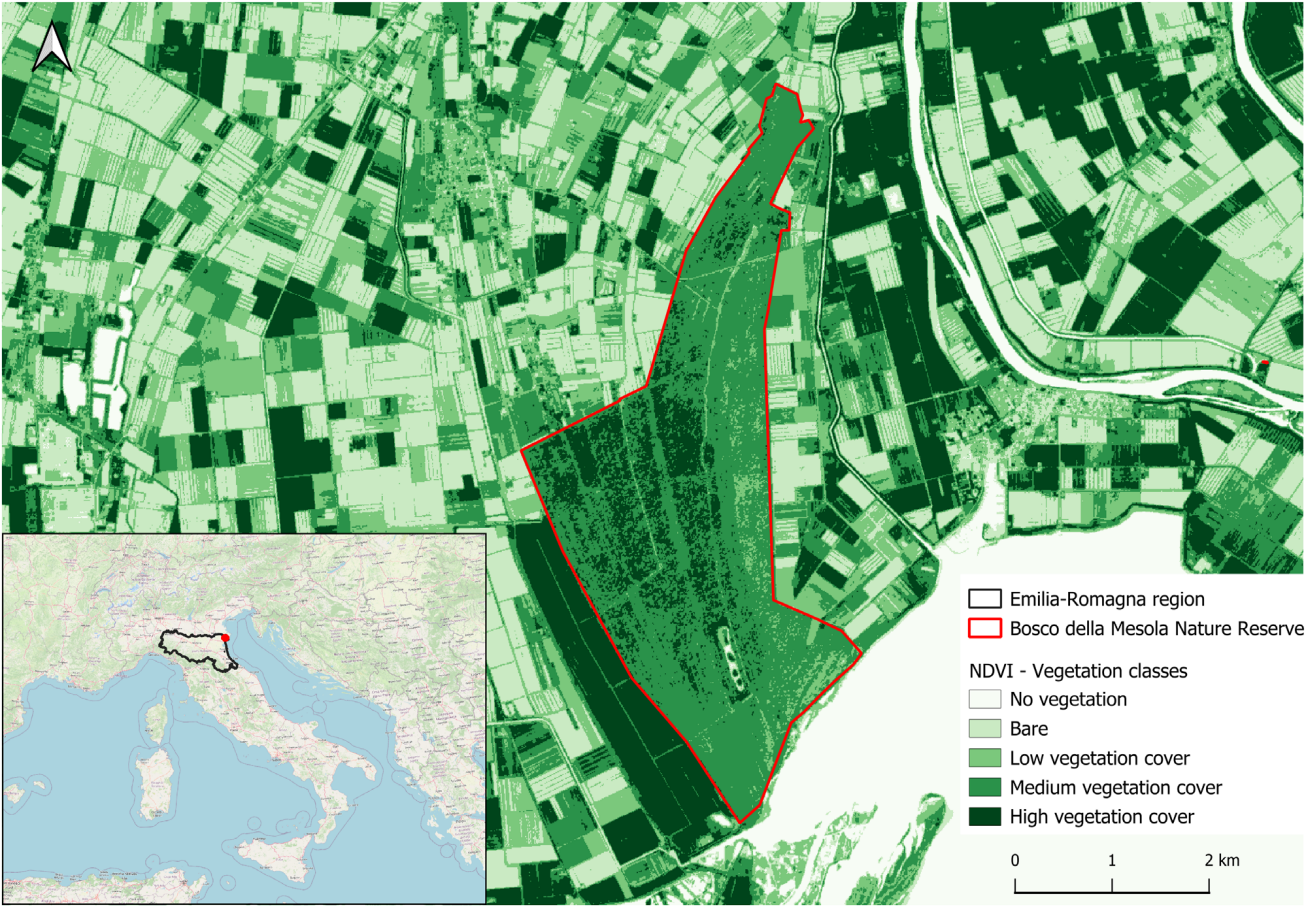
Bosco della Mesola Nature Reserve. NDVI=Normalised Difference Vegetation Index calculated using band 4 (red) and band 8 (near‐infra‐red) of the Copernicus Sentinel‐2 images (acquired in April 2024).

The Bosco della Mesola Nature Reserve is a lowland forest that grows on a system of dune ridges of very ancient origin located in the Emilia‐Romagna Region (Northern Italy, 44.8570°, 12.2556°) at the southern edge of the Po River Delta. This forest survived centuries of deforestation, transformations and territorial fragmentation. The soil is of alluvial origin and partly forms pools of water with marsh vegetation. In the early 1950s, when the forest was at risk of being converted into farmland, ownership was transferred to the State Forestry Agency. From that moment on, forest management focused on strengthening the forest's natural value. In 1958, the forest's perimeter was enclosed. In 1971, a 222 ha Integral Nature Reserve was established, and in 1977, the entire area was designated as a Nature Reserve (1058 ha) (Figure 1).

The Italian red deer population that lives in the Reserve is one of the few in Europe that has never been subject to restocking with specimens from external sources. Centuries of geographic isolation within a lowland coastal wood habitat have led to the development of an endemic subspecies, currently represented by a relict population of approximately 300 individuals (Zachos et al., 2014).

In addition to the Italian red deer, the only other ungulate present is the fallow deer (*Dama dama*).

The fallow deer was present in the area since the Renaissance, but it was exterminated around 1945 and later reintroduced between 1957 and 1965, likely using founders from the Presidential Estate of San Rossore, a vast alluvial plain in Tuscany, near Pisa, whose territory includes wetland areas, coastal sand dunes and floodplain forest (Lovari & Nobili, 2010; Mattioli et al., 2003). In the following decades, the fallow deer experienced a significant population increase, making it markedly more abundant than the Italian red deer, to the point of becoming highly limiting for it (Raganella Pelliccioni et al., 2013). For this reason, from the mid 1990s, interventions were initiated to control the fallow deer through a programme of capture and shooting; the programme continued until 2014 with a residual population of 10 individuals. Currently, approximately 80 fallow deer are estimated to be present in the Reserve according to the Forestry Police Corp of the Bosco della Mesola Nature Reserve (personal communication).

### Sampling

In March 2023 and October 2024, it was possible to perform an opportunistic sampling of ectoparasites from Italian red deer in the Bosco della Mesola Nature Reserve. The occasion was provided by the intention to safeguard this fragile population through a translocation initiative led in 2023 by WWF, Italy, and made possible through collaboration between various organisations and institutions. This involves relocating at least 20 individuals per year for three consecutive years to the Serre Regional Nature Park in Calabria, in order to establish a second wild‐living population of this endemic subspecies and reduce its risk of extinction.

Taking advantage of this opportunity, during the capture operations, hippoboscids were collected from the tegument of the sedated hosts. Twenty deer were examined in March 2023 and 32 animals in October 2024. The arthropods were preserved in a 70% ethanol solution.

Additionally, it was possible to examine *Lipoptena* specimens collected in 2007 from fallow deer during one of the culling programmes in the same Reserve. Veronesi et al. (2011) conducted a study on the prevalence of *Anaplasma phagocytophilum* in the fallow deer culled in 2007 and on the ticks collected from them. In addition to the ticks, several deer keds were also collected, which were not included in the mentioned study and remained unidentified in the parasitology laboratory of the University Department of some of the authors (DIMEVET). Morphological identification was based on the descriptions by Oboňa et al. (2022, 2023) and Salvetti et al. (2019). The description of *L. andaluciensis* by González et al. (2024) and the description of *L. fortisetosa* of Maa (1965) were also examined.

### Molecular analysis

Two keds from 2023/2024 and two from 2007 were cut longitudinally, and half of the body was placed in a 1.5 mL Eppendorf tube containing approximately 500 μL of TE buffer. The sample was then homogenised using a micro pestle and centrifuged at maximum speed to remove excess lipids and ethanol. The resulting pellet was used for DNA extraction, performed with the PureLink® Genomic DNA Mini Kit (Invitrogen, Thermo Fisher), following the manufacturer's protocol. A barcode fragment of 710 bp of COI gene was amplified using the universal LCO‐1490 forward primer and HCO‐2198 reverse primer (Folmer et al., 1994), under the reaction conditions and thermal profile described by Tuccia et al. (2016). PCR amplifications were conducted using a T‐personal thermal cycler (Biometra, Göttingen, Germany). The resulting PCR products were electrophoresed on a 1% agarose gel stained with SYBR Safe DNA Gel Stain (Thermo Fisher Scientific, Carlsbad, CA, USA) in 0.5× TBE buffer. Amplicons were excised from the gel, purified using the NucleoSpin Gel and PCR Cleanup kit (Mackerey‐Nagel, Düren, Germany), and sequenced on an ABI 3730 DNA analyser (StarSEQ, Mainz, Germany). Trace files were assembled using Contig Express (VectorNTI Advance 11 software, Invitrogen, Carlsbad, CA, USA), and consensus sequences were compared with available data using BLAST tools (https://blast.ncbi.nlm.nih.gov/Blast.cgi) (accessed on 15‐02‐2025). Sequence alignments were performed using BioEdit 7.2.5 (Hall, 1999), and the phylogenetic tree was inferred using the Maximum Likelihood method based on the General Time Reversible model (Nei & Kumar, 2000) with 1000 bootstrap replicates, implemented in MEGA7 software (Kumar et al., 2016). Accession numbers of obtained sequences are provided within the phylogenetic tree.

## RESULTS

**FIGURE 2:**
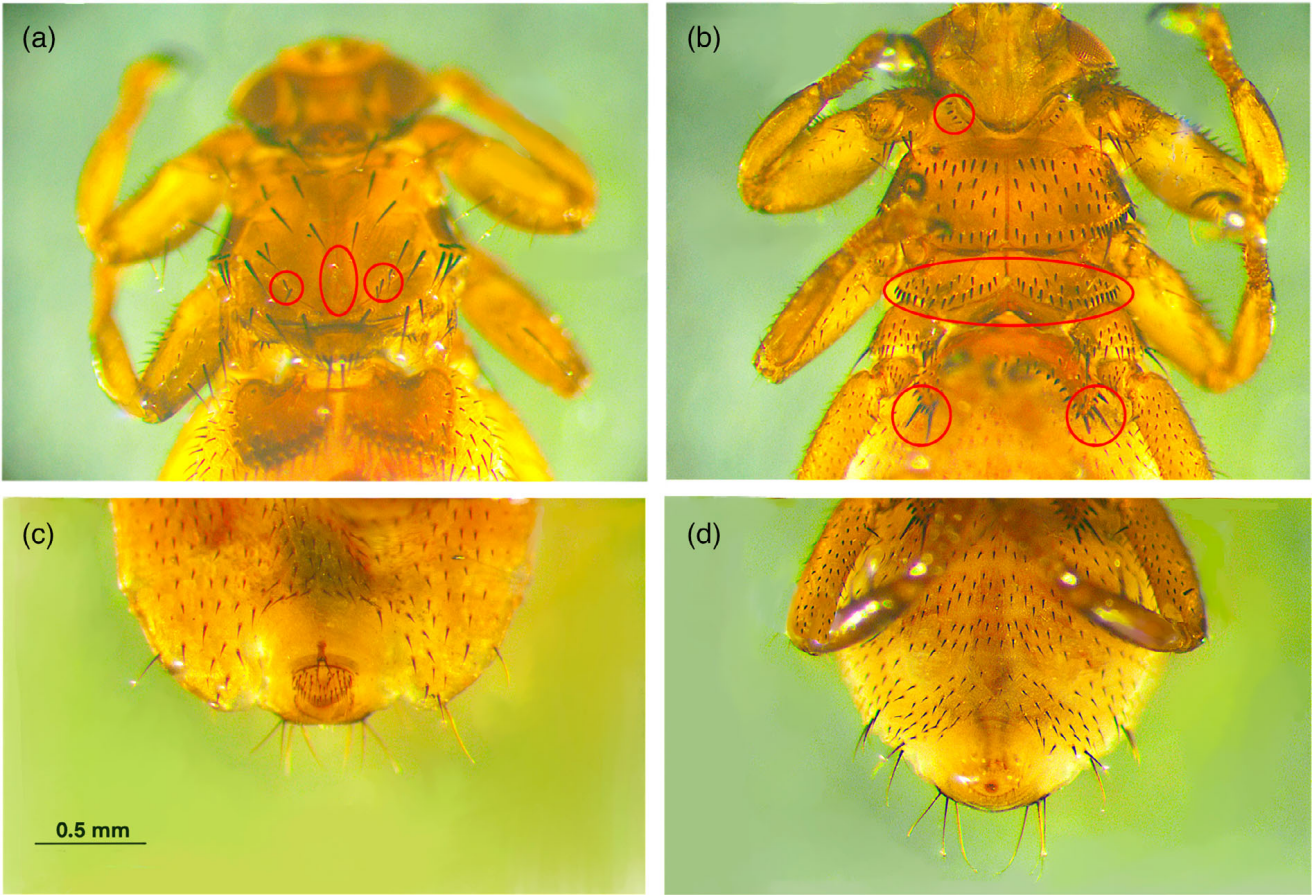
Morphological features of *Lipoptena andaluciensis*. A. chaetotaxy of the thoracic bristles: The red circles highlight the absence of acrostichal bristles and the presence of three small setae posteriorly in the scutum. B. ventral view: The red circles highlight the prosternal setae, the two rows of metasternal spines and the two bristles on the outer margin of the 1st abdominal sternite. C. female terminalia. D. male terminalia.

**FIGURE 3:**
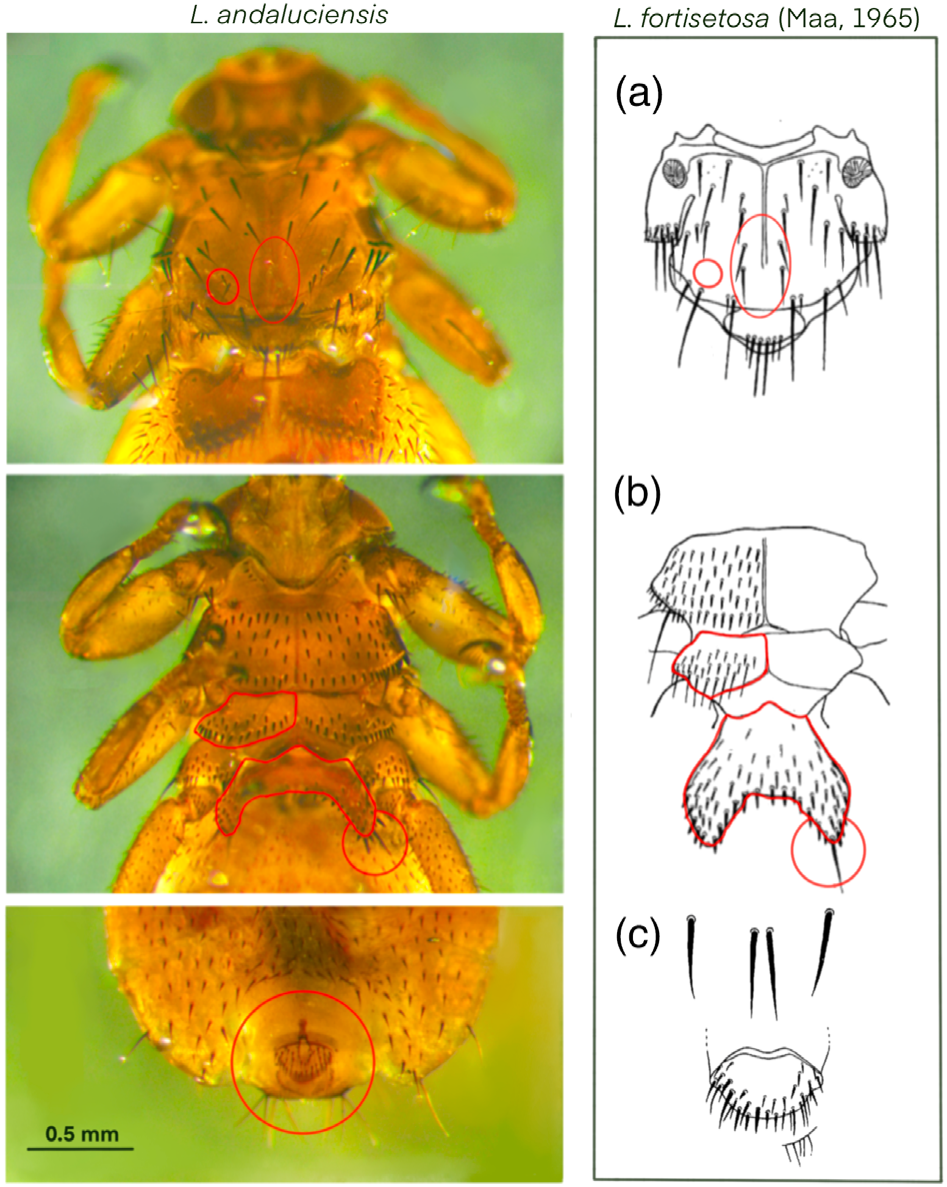
Main differences between *Lipoptena andaluciensis* female sampled in 2023 and *L. fortisetosa* as described by Maa (1965). A. thorax in dorsal view with the chaetotaxy of the bristles; B. ventral view with metasternum spines and 1st abdominal sternite highlighted in red; C. pregenital sclerite of the female.

All the individuals sampled in both 2023 (161 specimens, 74 males and 87 females from Italian red deer) and 2024 (96 specimens, 38 males and 58 females) are morphologically compatible with the description given by González et al. (2024) of *Lipoptena andaluciensis*, which differs from *L. fortisetosa* by the following characteristics (Figures 2, 3):

- Absence of acrostichal bristles on both sides of mediant notal suture, present, on the contrary, in *L. fortisetosa*;
- Presence of a group of 2–3 bristles on both sides of the posterior scutum, absent in *L. fortisetosa*;
- A lower number of prosternal setae (4–5) compared with *L. fortisetosa* (5–8);
- Two irregular rows of metasternum spines versus three rows in *L. fortisetosa*;
- Two bristles (one apical and one subapical) on each side of the outer margin of the first abdominal sternite versus one apical bristle in *L. Fortisetosa*;
- Absence of a pair of isolated setae near the pregenital sclerite, present in *L. fortisetosa*.

Along with the adults, 20 pupae were also collected in 2023 and 4 in 2024. Furthermore, in the samples from October 2024, there were also winged adults (3 females and 1 male), which were absent in the samples from March 2023.

Figure 3 highlights the main differences between a *L. andaluciensis* specimen collected in 2023 and *L. fortisetosa* as described by Maa (1965).

**FIGURE 4:**
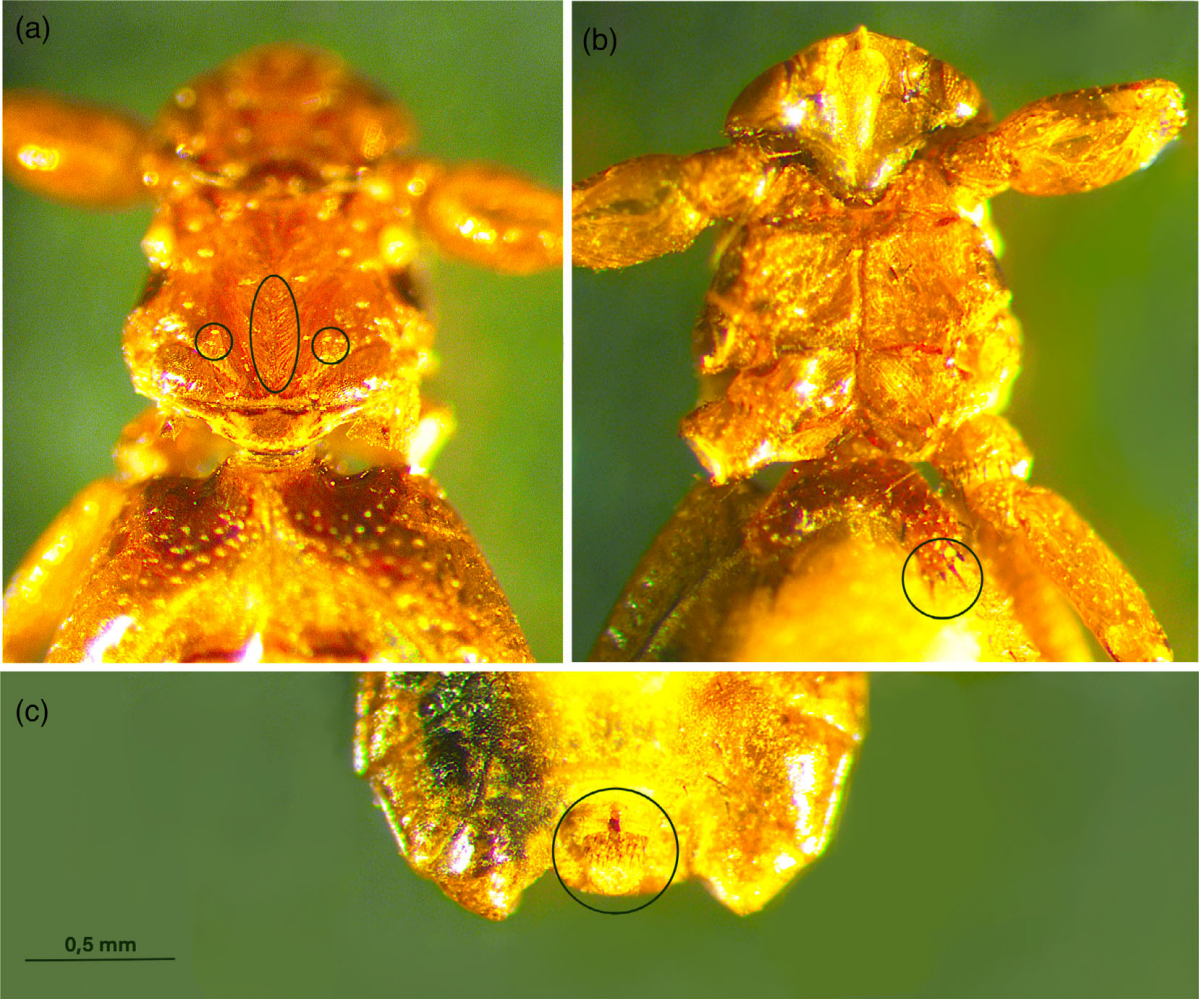
Morphological characteristics of *Lipoptena andaluciensis* sampled in 2007 on fallow deer. Although the specimen is in poor condition, the following features are clearly visible: A. the insertion points of the thoracic bristles; b. the two bristles on the 1st abdominal sternite; c. the pregenital sclerite of the female.

Despite the poor condition of dry‐preserved specimens collected from fallow deer in 2007, 66 *Lipoptena* specimens were examined and morphologically identified as 63 *L. andaluciensis* and 3 *L. cervi* (Figure 4).

**FIGURE 5:**
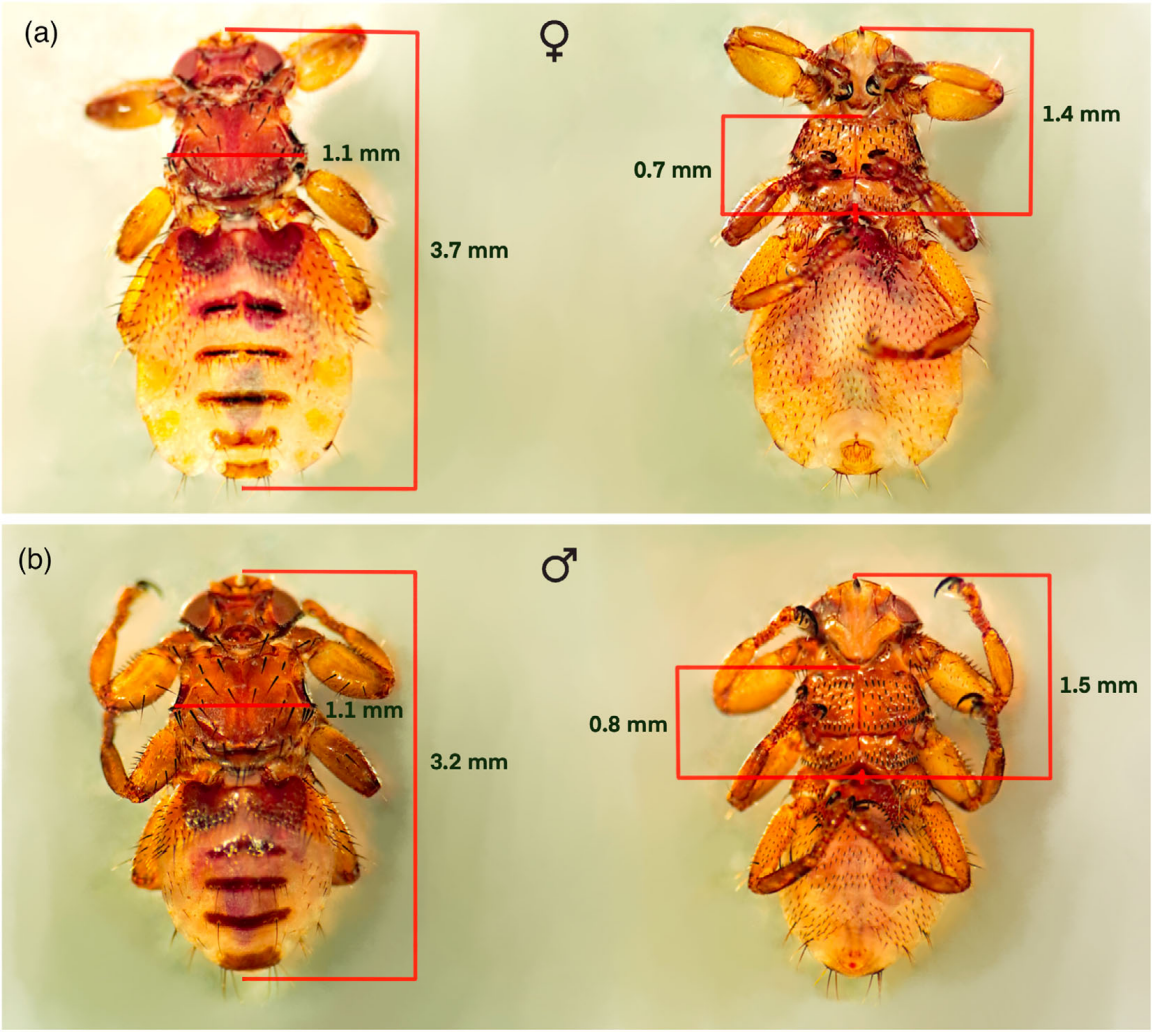
Dorsal and ventral view of *Lipoptena andaluciensis* with the main body measurements indicated. A. female. B. male.

The body length of *L. andaluciensis* specimens was, on average, 3.5 mm for females and 3 mm for males (minimum 2.5 mm for unfed specimens; maximum 4 mm for females with the larva in the abdomen), which is in line with those reported by González et al. (2024) for unfed winged adults. The maximum thorax width is approximately 1 mm for both males and females (Figure 5).

The body length of the 3 *L. cervi* specimens (2 females and 1 male) was around 5 mm; the maximum thorax width was 2 mm.

### Molecular identification

Due to their poor conditions, it was not possible to obtain sequences from the samples of 2007. *Lipoptena andaluciensis* specimens from 2023/2024, which were morphologically identical to each other, exhibited different BLAST identities when compared with sequences available in GenBank. Specifically, one of the two sequences (GenBank Accession Number PV291904) showed a 100% coverage and identity with PQ176810 and the other *Lipoptena andaluciensis* reference sequences reported by González et al. (2024). Conversely, the other sequence (GenBank Accession Number PV329828) despite the same query coverage, displayed lower similarity (approximately 95%) towards the same above‐mentioned reference sequences.

The two sequences obtained here, targeted by the standard Folmer's primers typically used in barcoding and metabarcoding projects, diverged one from the other for 35‐point mutations spread among the whole fragment (pairwise p‐distance 0.0534; supplementary Figure S1). In particular, sequence PV329828 showed 10 different amino acids, a nonsense codon due to a deletion at position 227 and a premature termination codon TAA as a consequence of two transitions in position 589 and 590 (C/T and G/A respectively), revealing the presence of a nuclear copy of the COI gene (a NUMT) in the *L. andaluciencis* nuclear genome. Despite this huge divergence, portions of the original COI protein can still be detected, being the 52.35% unambiguously aligned with the mitochondrial COI sequence.

**FIGURE 6:**
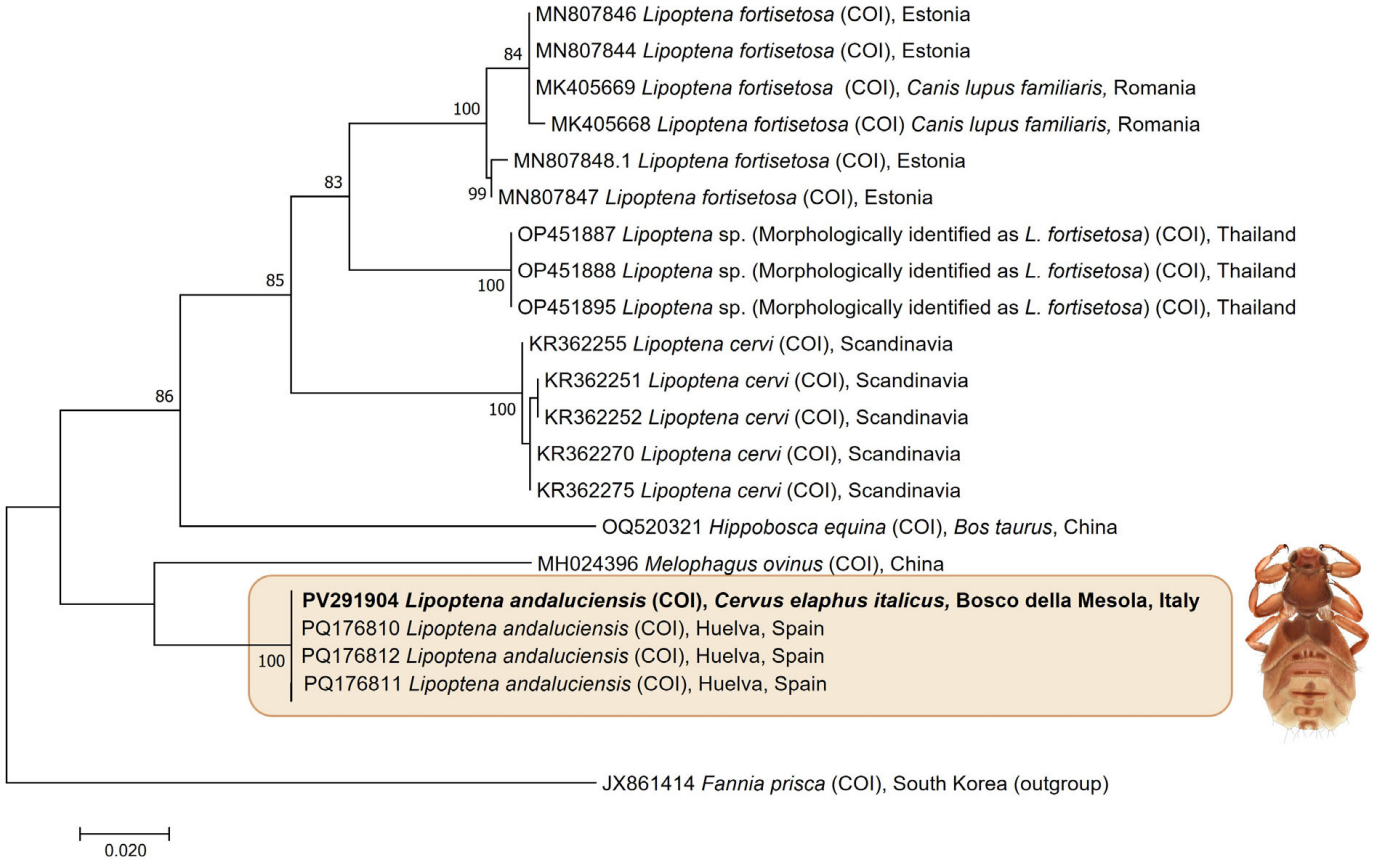
Maximum Likelihood phylogenetic tree. Bootstrap values, expressed in percentage higher than 75%, are provided near to the nodes. The tree is drawn to scale, with branch lengths measured in the number of substitutions per site. The analysis involved 22 nucleotide sequences. All positions containing gaps and missing data were eliminated. There were a total of 576 positions in the final dataset.

As regard the phylogenetic analysis, the sequence that matched *L. andaluciensis* sp. nov. with total identity (PV291904) clustered within a well‐supported group corresponding to this newly described species (Figure 6; bootstrap value 100). By contrast, the other sequence (PV329828) clustered apart yet remained within the broader clade comprising *L. andaluciensis* reference sequences, with a highly supported node (bootstrap value 100) as shown in supplementary Figure S2.

## DISCUSSION

The discovery of *Lipoptena andaluciensis* in the Bosco della Mesola Nature Reserve represents the first record of this species in Italy and in a peculiar environment such as a relict lowland forest confined inside an area with high anthropogenic impact. The climatic characteristics, in terms of average temperatures and precipitation, are typical of the Mediterranean climatic zone, similar to those of the region where this species was first reported by González et al. (2024).

Furthermore, the specimens collected from fallow deer in 2007 attest to the long‐term presence of this species in Italy, indicating a well‐established adaptation to our territory. While González et al. (2024) had only winged specimens captured using BG‐sentinel traps– thus lacking information about the host parasitised by this new species– we found *L. andaluciensis* on both red deer and fallow deer, suggesting a close association to deer species similar to the majority of *Lipoptena* species.

The *Lipoptena* species reported in Italy so far include the widespread and native *L. cervi* and the recently recorded allochthonous *L. fortisetosa* (Andreani et al., 2019). However, Andreani et al. (2019) provide identification images and descriptions highlighting only the specific features necessary to differentiate *L. fortisetosa* from the autochthonous *L. cervi*, without taking into account all the relevant taxonomic features described in Maa (1965) and considered in detail by González et al. (2024). Among the morphological traits discernible from the images published by Andreani et al. (2019), some of them—such as the details of the female pregenital sclerite and the thoracic bristle chaetotaxy—appear closer to *L. andaluciensis* than to *L. fortisetosa*, raising doubts about the accuracy of the identification. It is worth noting, however, that the average body size of the males and females collected by Andreani et al. (2019) is larger than our specimens and those reported by González et al. (2024). Unfortunately, our haplotypes are not directly comparable with Andreani et al. (2020) because of the lack of their sequences on public repository, although they reported a 100% genetic identity with one sequence of *L. fortisetosa* isolated in Korea (GenBank Accession Number KU366895). It is possible, therefore, given some discrepancies between the morphologic and genetic features, that deer keds previously described in Italy comprise, besides *L. cervi*, both *L. fortisetosa* and *L. andaluciensis*.

In a very recent study by Stancampiano et al. (2025), based on data and identifications from Andreani et al. (2021), the geographical distribution of *L. fortisetosa* and *L. cervi* is analysed, along with the separation of infrapopulations of both species on the same host. This study highlights how the environmental preferences of *L. fortisetosa* differ from those of *L. cervi*: *L. fortisetosa* is found at low altitudes near urbanised areas, whereas *L. cervi* is distributed at higher elevations. The bioecological characteristics observed by Stancampiano et al. (2025) for the keds identified as *L. fortisetosa* species by Andreani et al. (2021) would be compatible with *L. andaluciensis*, a Mediterranean species found so far only at low altitudes.

In light of the discovery of *L. andaluciensis* in the Bosco della Mesola Nature Reserve, a morphological and genetic re‐examination of the *Lipoptena* specimens collected by Andreani et al. (2019, 2020, 2021) and further analysed by Stancampiano et al. (2025) would be beneficial.

The morphology of our specimens perfectly matches those provided by González et al. (2024), and the molecular identification supports their classification as *L. andaluciencis*. However, the discovery of NUMTs in the genome of *L. andaluciencis* suggests caution when using DNA‐barcoding as a unique tool for specimen identification. NUMTs, in fact, can have a serious impact on species identifications, phylogenetic reconstructions, and population studies, especially when they are located in the COI gene, the most frequently used molecular marker for several classes of Arthropoda (Buhay, 2009; Ožana et al., 2022). NUMTs are well documented in insects (Black IV & Bernhardt, 2009) and in the Hippoboscidae family as well (Šochová et al., 2017). Their presence can lead to erroneous identifications by inflating the apparent species richness (Hebert et al., 2023), further complicating the already complex phylogenetic framework of genus *Lipoptena*, which appears paraphyletic— that is, it includes the most recent common ancestor but not all of its descendants. In this scenario, a taxonomic revision of the Hippoboscidae family, paying attention to NUMTs, is therefore warranted. The analysis of additional nuclear and mitochondrial markers, together with COI, would be a solution.

Nothing is known regarding the epidemiology of this new species. The finding of *L. andaluciensis* in the Bosco della Mesola Nature Reserve is, in fact, enigmatic, and it demonstrates not only the presence of an unknown species in Italian territory but also a species that has been present for almost 20 years going unnoticed. Furthermore, it is intriguing to question where *L. andaluciensis* originates and to investigate its geographical distribution. Up to now, this species has been reported in two distant countries, but in similar forested Mediterranean habitat at low altitude. It would be interesting in this regard to conduct deer ked sampling also in other similar areas. For example, San Rossore Reserve and the Bosco della Mesola Nature Reserve have similar environmental characteristics: a lowland wood near the sea. Although almost nothing is known about the bioecology *of L. andaluciensis*, it is likely that this species has different environmental preferences compared with *L. cervi* that is more frequent over 600 m of altitude, similar to the questionable Italian *L. fortisetosa* (Stancampiano et al., 2025).

## AUTHOR CONTRIBUTIONS

**Federica Usai:** Writing – original draft; writing – review and editing; investigation; methodology; visualization. **Filippo Maria Dini:** Investigation; writing – review and editing; writing – original draft; visualization. **Ilaria Guarniero:** Methodology; investigation; writing – original draft; writing – review and editing; visualization. **Enrica Bellinello:** Investigation; resources; writing – review and editing. **Laura Stancampiano:** Conceptualization; methodology; writing – original draft; writing – review and editing; supervision; visualization.

## CONFLICT OF INTEREST STATEMENT

The data generated during the present study are available in the supplementary material and at NCBI GenBank https://www.ncbi.nlm.nih.gov under accession numbers PV291904, PV329828.

## ETHICS STATEMENT

Deer capture and handling was authorised by Raggruppamento Carabinieri Biodiversità, management body of the Natural State Reserve, under Permit Number 84/13–4/2022 signed on 17 January 2023, following the procedures reported in the feasibility study by ISPRA (National Institute for Environmental Protection and Research) on 9 December 2021.

## Supporting information


**Figure S1.** COI and NUMT pairwise alignment. Single point mutations are highlighted. In particular, the deletion in position 227 of NUMT sequence modifies the downstream reading frame (first new codon: GAG, glutamic acid).

**Figure S2.** Maximum likelihood phylogenetic tree comprising the NUMT sequence. Notice that this sequence clusters apart yet remaining in the *L. andaluciensis* clade. Bootstrap values, expressed in percentage higher than 75%, are provided near to the nodes. The tree is drawn to scale, with branch lengths measured in the number of substitutions per site.

## Data Availability

The data generated during the present study are available in the supplementary material and at NCBI GenBank https://www.ncbi.nlm.nih.gov under accession numbers PV291904, PV329828.
