# Supplementary figures and images for: The enigmatic case of Lipoptena sp. in the Bosco della Mesola Nature Reserve (Italy)
Source: Med Vet Entomol. 2025 Aug 20;40(1):82–90. doi: 10.1111/mve.70002 (PMC12865754; doi:10.1111/mve.70002)

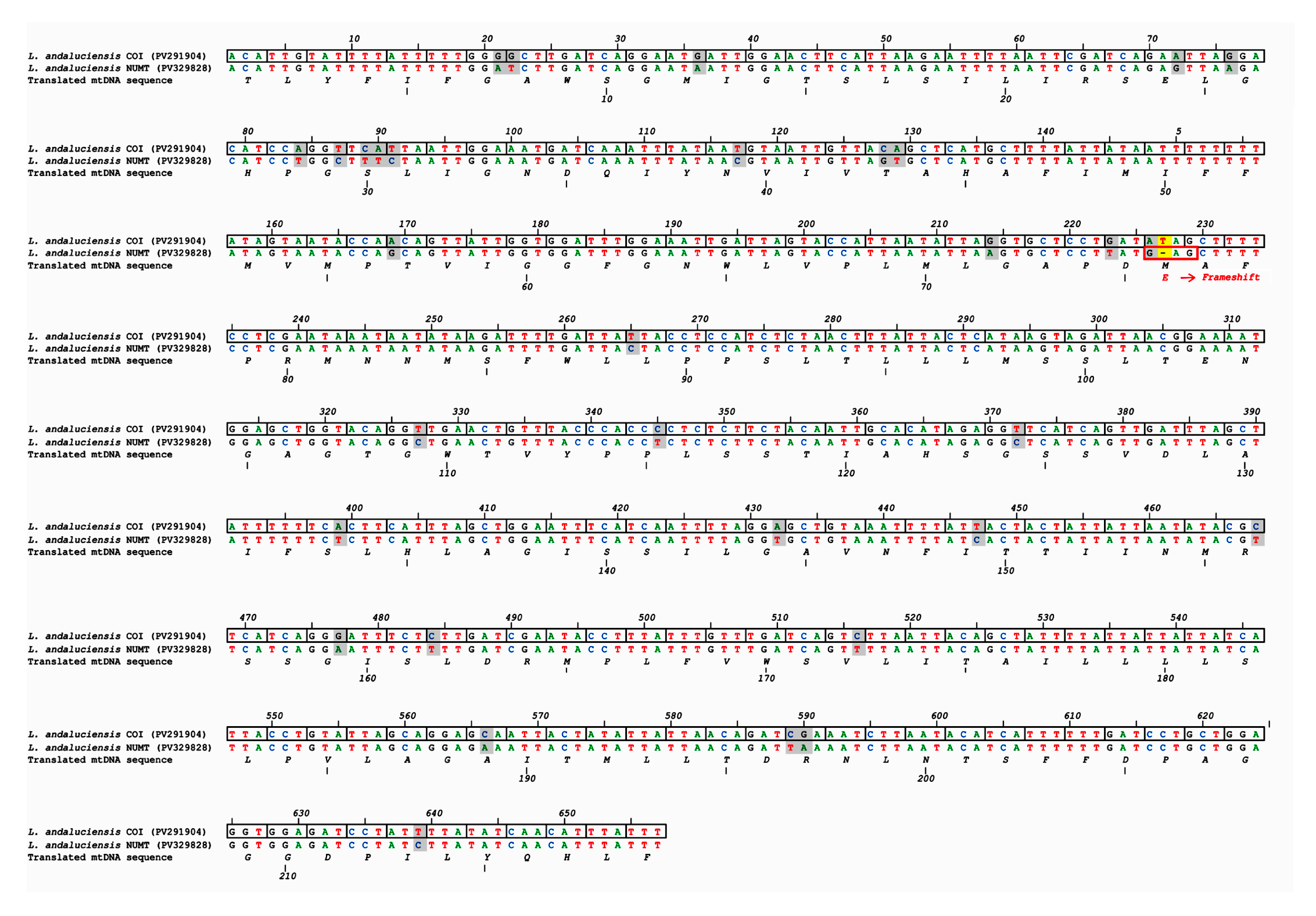

Supplement: Supplementary file 1 — Figure S1. COI and NUMT pairwise alignment. Single point mutations are highlighted. In particular, the deletion in position 227 of NUMT sequence modifies the downstream reading frame (first new codon: GAG, glutamic acid). [file MVE-40-82-s002.tiff]

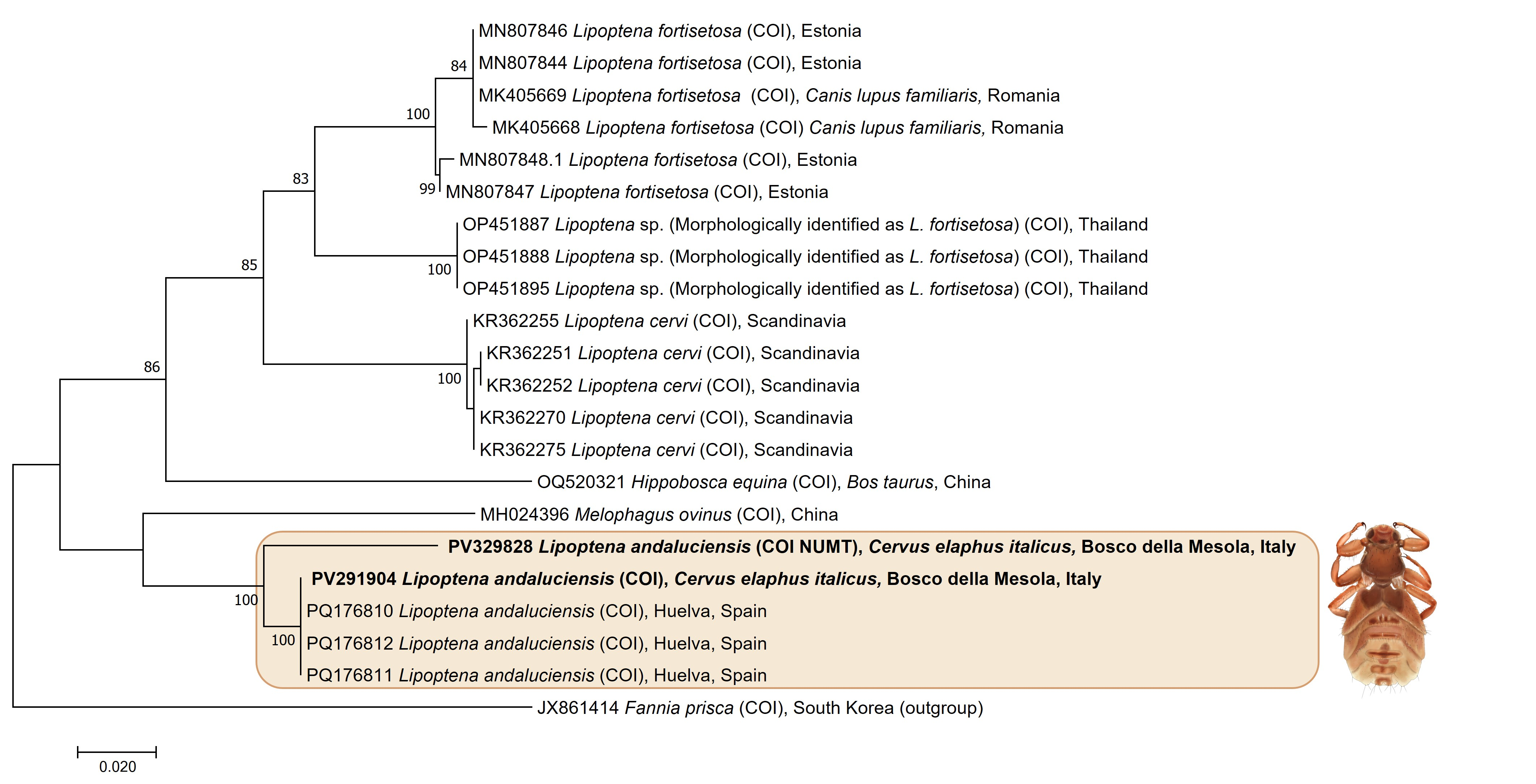

Supplement: Supplementary file 2 — Figure S2. Maximum likelihood phylogenetic tree comprising the NUMT sequence. Notice that this sequence clusters apart yet remaining in the L. andaluciensis clade. Bootstrap values, expressed in percentage higher than 75%, are provided near to the nodes. The tree is drawn to scale, with branch lengths measured in the number of substitutions per site. [file MVE-40-82-s001.tiff]
